# The development and maintenance of immunity against visceral leishmaniasis

**DOI:** 10.3389/fimmu.2024.1486407

**Published:** 2024-12-09

**Authors:** Rahul Tiwari, Awnish Kumar, Vishal Kumar Singh, Rajneesh, Shashi Bhushan Chauhan, Shyam Sundar, Susanne Nylén, Christian Engwerda, Rajiv Kumar

**Affiliations:** ^1^ Centre of Experimental Medicine and Surgery, Institute of Medical Sciences, Banaras Hindu University, Varanasi, India; ^2^ Department of Medicine, Institute of Medical Sciences, Banaras Hindu University, Varanasi, India; ^3^ Department of Microbiology Tumor and Cell Biology, Karolinska Institutet, Stockholm, Sweden; ^4^ Infection and Inflammation Division, QIMR Berghofer Medical Research Institute, Brisbane, QLD, Australia

**Keywords:** leishmaniasis, immunological memory, CD4 + T cell responses, effector T cells, tissue resident memory T cells, vaccines

## Abstract

Understanding the development and maintenance of immunological memory is important for efforts to eliminate parasitic diseases like leishmaniasis. Leishmaniasis encompasses a range of pathologies, resulting from infection with protozoan parasites belonging to the subgenera *Leishmania* and *Viannia* of the genus *Leishmania.* A striking feature of these infections is that natural or drug-mediated cure of infection generally confers life-long protection against disease. The generation of protective T cell responses are necessary to control *Leishmania* infections. CD4^+^ T helper (Th) cells orchestrate immune responses in leishmaniasis and IFNγ^+^ Tbet^+^ CD4^+^ T (Th1) cells are required for the activation of phagocytes to kill captured or resident parasites, while other Th cell subset, including FoxP3^+^ natural regulatory T cells and Th2 cells can promote disease progression by suppressing the activities of Th1 cells. Upon resolution of a primary *Leishmania* infection, different subsets of CD4^+^ T cells, including tissue-resident memory T cells, effector memory T cells, central memory T cells, and short-lived effector T cells, help to confer resistance against reinfection. To maintain long-term protective *Leishmania-*specific CD4^+^ T cells responses, it is believed that persistent parasites or re-exposure to parasites at regular intervals is required (concomitant immunity). Despite the advances in our understanding about the immune responses during leishmaniasis, the generation of long-lasting protective immunity via vaccination has yet to be achieved. In this review, we summarize our current understanding about the formation and maintenance of immunological memory and control of leishmaniasis at the individual and population level. We will focus on Indian visceral leishmaniasis and discuss T cell responses that contribute to susceptibility to leishmaniasis, parasite persistence in populations and the environment, as well as describing advances in the development of leishmaniasis vaccines aimed at inducing protective CD4^+^ T cell responses.

## Introduction

Leishmaniasis represents a diverse array of clinical manifestations resulting from infection with intracellular protozoan parasites of the subgenera *Leishmania* and *Viannia* of genus *Leishmania*. Parasites are transmitted to humans through the bite of infected sand fly vectors belonging to the genus *Phlebotomus* in the Old World (Africa, Asia, and Southern Europe) and the genus *Lutzomia* in the New World (The Americas). Disease symptoms range from relatively benign lesions in cutaneous leishmaniasis (CL), to mucosal membrane destruction in mucocutaneous leishmaniasis (MCL), to lethal infection of visceral organs in visceral leishmaniasis (VL), also known as Kala-azar in the Indian sub-continent. The different disease pathologies depend primarily on the parasite species involved, but also on the type of host to immune response generated following infection (1). With an extremely varied epidemiology, leishmaniasis is endemic in 99 nations, and 9 of these are endemic for VL alone, 9 for CL, and 71 for both VL and CL (https://www.who.int/publications/i/item/who-wer9840-471-487). CL, the most prevalent form of the disease, presents as skin lesions, predominantly ulcers, typically on exposed areas of the body. These lesions can lead to significant disabilities and may result in lifelong scarring (1, 2). Approximately 95% of CL cases are reported in the Americas, the Mediterranean basin, the Middle East, and central Asia. It is estimated that between 600,000 to 1 million new cases of CL are reported worldwide annually. VL is the most severe form of leishmaniasis and is caused by *Leishmania (Leishmania) donovani* (in Asia), *Leishmania (Leishmania) infantum* (South America and around the Mediterranean basin) and *Leishmania donovani* complex (in Africa) (3). VL continues to pose a substantial public health challenge in numerous countries, with the majority of reported cases originating from Brazil, east Africa, and India, particularly affecting socioeconomically disadvantaged people With an estimated annual disease burden of 50,000 to 90,000 cases, the World Health Organization (WHO) considers VL as a disease of outbreak potential, (Leishmaniasis, 12 January, 2023, https://www.who.int/news-room/fact-sheets/detail/leishmaniasis). In VL patients, visceral organs are parasitized and clinical manifestations are systemic, including long-term fever, enlargement of the spleen and liver, weight loss, progressive anemia, pancytopenia and elevated antibody titers (4). Lymphadenopathy and skin darkening can also be observed if the disease is allowed to progress. Comorbidities in VL patients can include concomitant bacterial and HIV infections, which can affect clinical presentations and impair the accuracy of early diagnostic tests (5, 6). These are important because failed diagnosis can have severe consequences as fatality rate is over 95% unless VL is treated (4, 7). It is important to understand that *Leishmania* infections do not always result in disease and that the majority of infected individuals remain asymptomatic with no clinical signs (5). Factors influencing these different outcomes of infection include host genetics (6), nutritional status (5), and co-infections (8, 9), which affect host immune responses.

The reservoir host for the *Leishmania* parasite exhibits regional variation, and both anthroponotic and zoonotic transmission of leishmaniasis can occur. Zoonotic transmission is considered to dominate in Mediterranean regions and South American endemic foci, while VL is predominantly anthroponotic in India and Bangladesh (10–12). Infection begins when a carrier sand fly lacerates a blood vessel and feeds on the pool of blood released. During this process, the fly releases a mixture of salivary factors with anticoagulant, vasodilatory, and complement inhibitory activities. As the sand fly feeds, it regurgitates and transmits the infectious motile metacyclic promastigotes into the mammalian host (13). Inside the host, promastigotes convert into non-motile amastigotes, that take up permanent residence in macrophages of tissues, which in case of VL is the spleen, liver and bone marrow (14). VL presents as complex immunological responses involving both innate and adaptive responses. Experimental infections have demonstrated that the first immune cells to respond to the insult of an infectious bite are infiltrating neutrophils followed by monocytes (15–17). These cells can kill the parasites if activated but can also act as host cells allowing the establishment of disease. Macrophages, and in particular dendritic cells (DC), are involved in activating the adaptive immune response and shaping it through the cytokines secreted when these antigen presenting cells (APC) are in contact with T cells. These interactions shape the T cell immune response and determine disease outcome.

Concomitant immunity provided by memory T cells is thought to develop following primary infection with *Leishmania* parasites, and complete parasite elimination can impair protection against reinfection (18, 19). Memory T cells can develop at different time following infection and have varying life spans and functions (20). Defining the processes underlying the generation of effective immune memory responses is needed to help design vaccine approaches that produce long-lasting cell-mediated immunity. However, despite advances in our understanding of these processes, significant gaps remain in our understanding of how memory T cells are generated, and in particular, how to generate these responses by vaccination.

## T cell memory response

Immunological memory is a feature of the adaptive immune system and includes the development of long-lived memory T cell and B cells (along with long lived plasma cells) and their different subsets, either located in secondary lymphoid organs (SLOs) or in peripheral tissue (21). As the primary immune response contracts, only around 5-10% of antigen-specific T cells survive and become memory T cells. These cells have less rigorous requirements for re-activation and can respond to suboptimal T cell receptor (TCR) and co-stimulatory signaling with greater proliferative and effector capacity, as well as tissue homing capabilities, compared with naïve T cells (reviewed in (22). Commitment of antigen-specific T cells to effector or memory lineages is determined by priming conditions, such as the duration and strength of antigenic exposer or stimulation, co-stimulatory signals, and local cytokine production (23–25). Memory T cells were first identified as CD45RO^+^and CD45RA^-^ in humans (26) and were further divided into central memory cells (Tcm) and effector memory cells (Tem) on the basis of CCR7 and CD62L expression in mice and humans, which are constitutively expressed on naïve T cells enabling their localization in secondary lymphoid organs (SLO). Tcm are highly proliferative, interleukin (IL)-2 secreting CD45RA^-^CCR7^+^CD62L^+^ cells present in SLO, whereas Tem is efficient effector cytokine producing CD45RA^-^CCR7^-^CD62L^-^ cells present in peripheral tissue and recirculate via the blood. A more recently described memory subset is tissue resident memory T (Trm) cell and is important as a first line of defense following re-exposure to a pathogen in non-lymphoid tissues. Trm cells express high levels of CD69 and lower levels of CD62L, facilitating their tissue homing and retention capacity (21, 27). Although memory T cells respond rapidly to parasites following re-exposure, little is known about their persistence after cure of visceral disease, despite numerous studies (28, 29).

## T cell responses in leishmaniasis

Early studies of *Leishmania* infection in mice helped develop the paradigm for T helper cell differentiation and committed effector functions (30). A central cytokine produced by DC in priming protective immune responses is IL-12. Production of IL-12 during CD4^+^ T cell priming drives the generation of IFN-γ producing Th1 cells that are essential for macrophage activation and control of *Leishmania* parasites (31, 32). The CD4^+^ T cell responses seen in leishmaniasis patients depend on the causative species and the site of infection. In VL, which is a progressive systemic disease, patients present a highly mixed immune profile, where pro-inflammatory mediators like IL-1, IL-6, IL-8, IL-12, IL-15, IFN-γ and TNF-Α, along with IL-10 and IL-4 are increased in the plasma (33, 34). In spleen, liver and bone marrow, CD4^+^ T cells express elevated levels of IFN-γ along with IL-10 (34) (reviewed in (35). Interestingly, VL does not lead to the expansion of FoxP3^+^ Treg cells (36). Instead, chronic infection with *L. (L.) donovani* is associated with the development of type I regulatory (Tr1) cells, which were defined as IL-10 producing-CD4^+^CD25^-^Foxp3^-^ T cells in VL patients (34). Subsequent studies in mice showed that the transcription factor Blimp1 was crucial for Tr1 cell development and IL-10 production (37). These Tr1 cells are believed to develop from Th1 cells and suppress pro-inflammatory immune responses, serving to protect the patient from tissue pathology (38). However, by doing so, they may also promote parasite survival. In mice infected with *L. donovani*, a distinct population of LAG3^+^CXCR5^+^ CD4^+^ T cells have also been observed recently. This subset is thought to play a role in the maintenance of T cell responses. These cells can differentiate into regulatory and effector cells and can confer protection on adoptive transfer (39).

**Figure 1:**
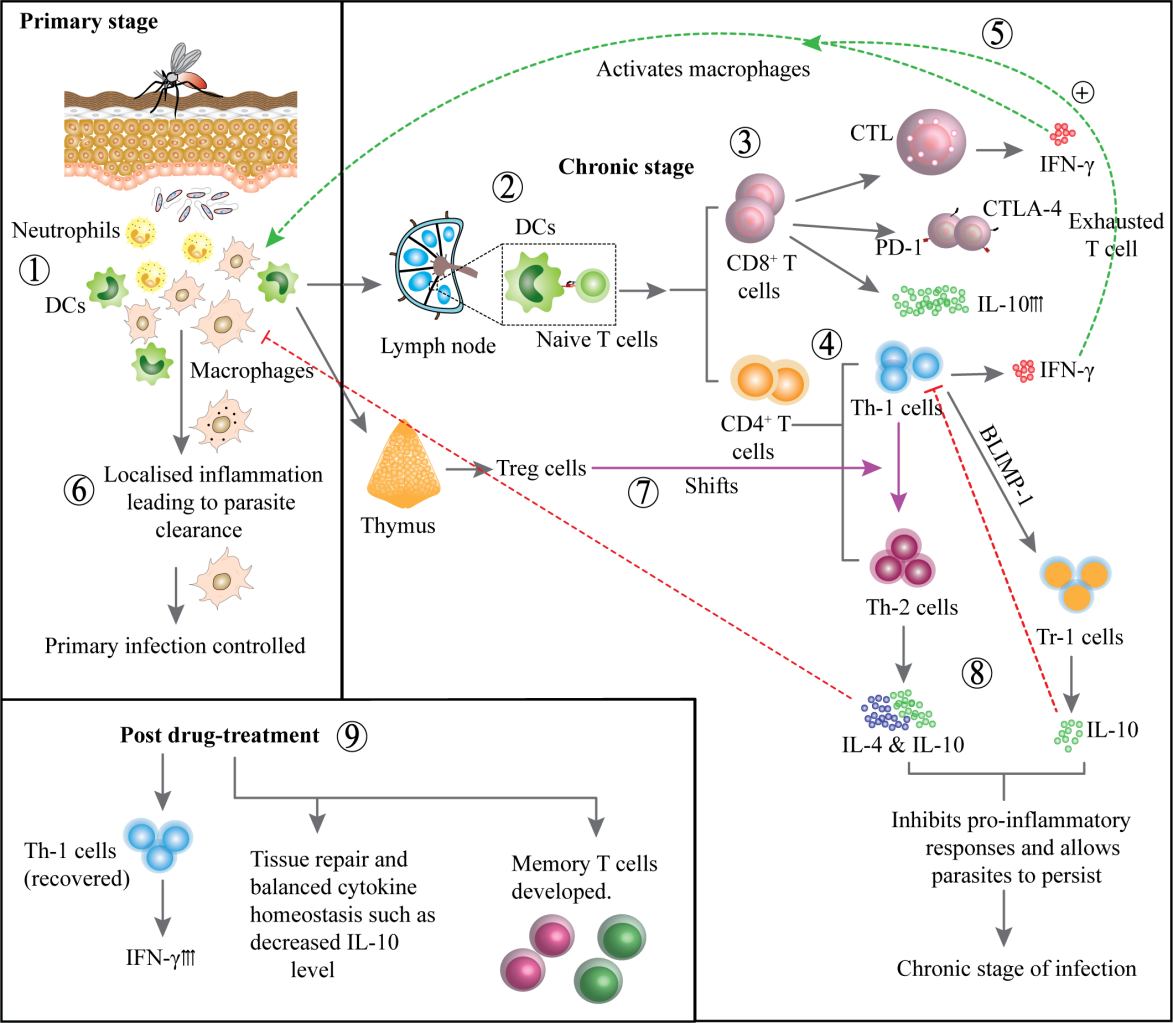
Development of immune responses during primary and chronic stages and post treatment: Neutrophils and DCs are activated at the infection site, initiating the immune response (1). DCs migrate to lymph nodes to present antigens to naïve T cells (2). CD8^+^ T cells become activated but may display exhaustion markers CTLA-4, PD-1 and produce IL-10 (3). CD4^+^ T cells differentiate into Th1 cells, that produces IFN-γ to activate macrophages (4). IFN-γ production enhances macrophage activation, contributing to parasite control (5). Primary infection is controlled by intracellular parasite clearance and localized inflammation (6). In contrast, Treg cells may suppress immune responses, promoting parasite persistence (7). T helper cells may shift towards Th2 phenotype producing IL-4 and IL-10 or Th1 can gradually develop into Tr-1 cells producing IL-10, inhibiting pro-inflammatory responses and promoting chronic infection (8). Following treatment, Th1 cell activity is restored, IFN-γ production increases, cytokine balance is re-established and memory T cells develop to support long term immunity (9).

CD8^+^ T cells in human leishmaniasis can play both protective and exacerbating roles, depending on their functional characteristics and state (40). On one hand, an IFNγ-dominant CD8^+^ T cell response is suggested to contribute to protective immunity in both murine and human VL, potentially aiding in control of the infection (40). Conversely, upregulation of IL-10 within CD8^+^ T cells has been associated with VL progression (41, 42). These cells can exhibit cytotoxic T lymphocyte (CTL) features, which are associated with increased IFN-γ production in plasma. However, CD8^+^ T cells in chronic leishmaniasis often also display markers of exhaustion or anergy, such as elevated expression of inhibitory receptors CTLA-4 and PD-1, which can impair their responsiveness to *Leishmania* antigens, as seen in whole blood assays and PBMC cultures (42, 43). Following successful treatment, CD8^+^ T cells can regain responsiveness, potentially providing protection against re-infection (42) (**Figure 1**). Additionally, experimental VL models indicate that blocking the B7-H1 (PD-1 ligand) pathway can reduce splenic parasite burden and alleviate CD8+ T cell exhaustion and apoptosis (44).

The lack of leptins, which is a feature of malnourished individuals and VL patients (45) has been suggested as a reason for the impaired and exhausted T cell phenotype seen in experimental VL (46). Experimental studies in mice show that CD8^+^ T cells contribute to the protective immunity. Murray et al. found that infection promoted the generation of CD8^+^ T cells that could protect against re-infection in experimental VL (47). Furthermore, a vaccine-induced CD8^+^ T cell response could confer protection against *L. (L.) donovani* in mice (48). CD8^+^ T cells can mediate protection via the secretion of IFN-γ, perforin and granzymes (49), and depleting CD8^+^ T cells was found to impair hepatic granuloma formation and was associated with a failure to curb liver infection in *L. (L.) donovani* infected mice (50). In human VL, Th17 cells have been associated with protection against *L. (L.) donovani* infection by mediating intracellular pathogen clearance along with Th1 cytokines (51). Moreover, experimental sensitization with live attenuated *L. (L.) donovani* has been reported to promote an efficient IL-17-dominant immune response that confers protection to the host (52) suggesting role for Th17 cells in protective memory responses.

## Generation and maintenance of CD4^+^ T cell memory responses in VL

Both CD8^+^ and CD4^+^ memory T cells generated after natural infection or vaccination are important for parasite control. But the generation and maintenance of these memory cells differ significantly. The generation and maintenance of CD4^+^ memory T cells likely requires persistence of parasite antigen or frequent re-exposure to parasites (18), while CD8^+^ memory T cells can be maintained without antigen persistence (53). However, there are some reports suggesting that CD4^+^ memory T cells can also be maintained after antigen clearance, but only when there was limited competition from other T cell clones (54, 55). A recent study from Brazil reported protective roles of Tcm and Tem positively correlated with IL-2, IFN-γ and TNF production by these cells in VL patients following drug treatment (56). These results indicate CD4^+^ memory T cell generation, but how long it was maintained and the role of these cells following reinfection needs further evaluation. However, a previous study evaluating cell mediated immune response in terms of lymphoproliferation and activation upon *in vitro* stimulation with leishmanial antigens in active VL, cured VL, and naive groups showed significantly high lymphoproliferation in cured VL group and a significant proportion of CD4^+^ and CD8^+^ T cells expressed activation markers. Individuals with a history of VL ranging from one to twenty years were included in this study, suggesting the presence of circulating memory T cells specific to *Leishmania*. A predominant Th1 type cytokine response was also observed (49). In line with the above findings another similar study reported maintenance of CD4^+^ T cells memory after recovery from VL. Interestingly, even leishmania skin test positive individuals showed similar memory T cell response on *in vivo* exposer to leishmanial antigens suggesting even asymptomatic infection can provide long term protection (57).

**Figure 2:**
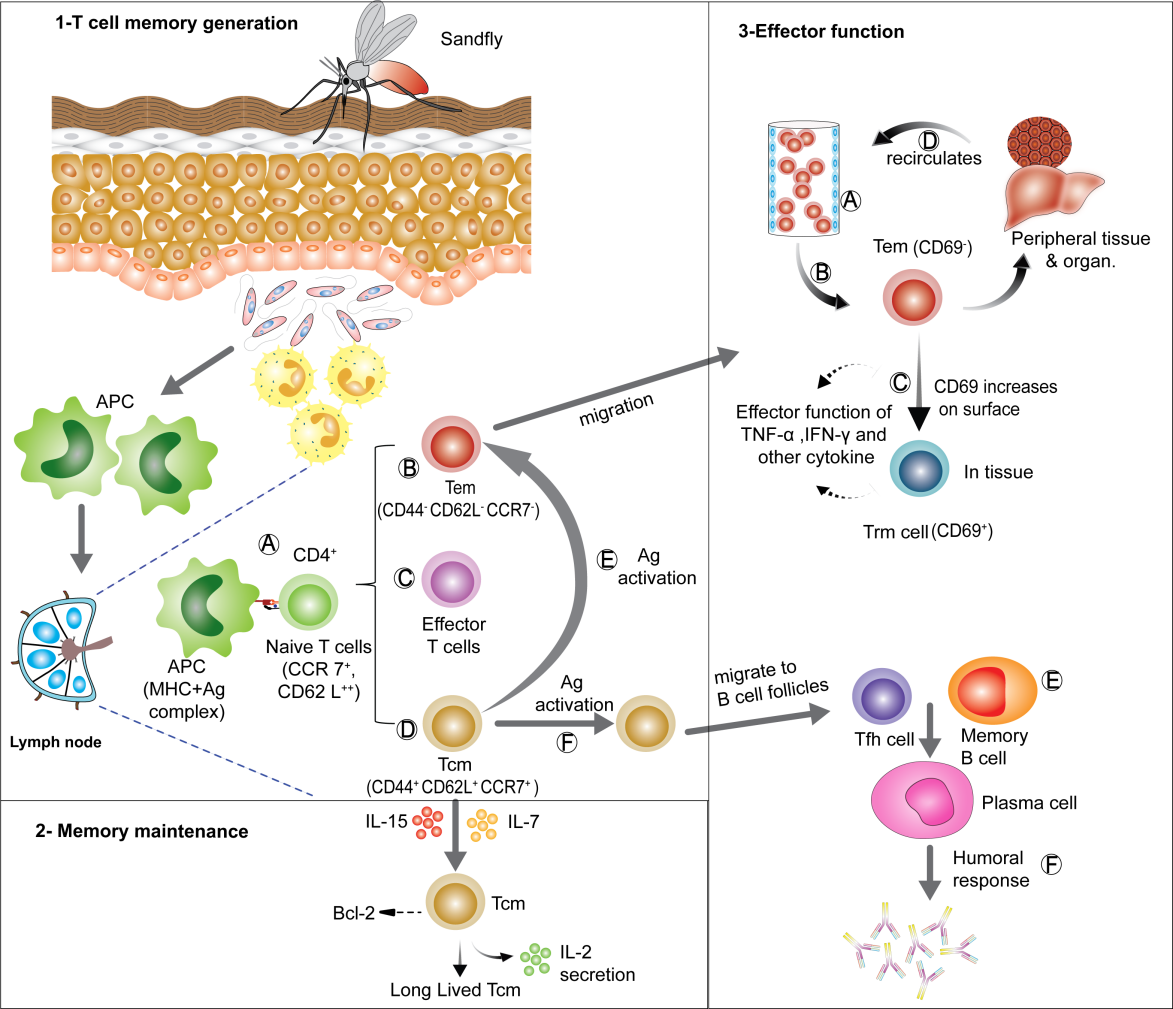
Generation, maintenance and effector functions of CD4^+^ T cell memory: (1) T cell memory generation- antigen presenting cells (APCs) take up parasites or parasite- infected neutrophils and moves to local lymph node for antigen presentation to naïve T cells. On recognizing the MHC II-peptide complex presented by APCs, naïve T cells get activated **(A)** and can differentiate into various subsets, such as Tem **(B)**, short lived effector T cells **(C)** and Tcm **(D)**. On re-exposer to antigen Tcm can re-differentiate into Tem **(E)** and Tfh **(F)**. (2) Memory maintenance- Tcm cells can be long-lived in lymphoid organs in presence of IL-7 and IL-15 signaling, which sustains anti-apoptotic Bcl-6 expression. (3) Effector functions- Tem can migrate via blood **(A)** to various tissues and organs performing effector functions **(B)**, Tem can also start expressing CD69 and can convert into tissue resident memory (Trm) cells, which can then act as a first line of defense on Ag re-exposer **(C)**. Tem can also migrate through various organs and tissues and scan them for Ag **(D)**. Tfh generated from Tcm cells can provide help to memory B cells **(E)** assisting early humoral (antibody) responses **(F)**. [APCs, Antigen presenting cells; Ag, Antigen; Tcm, central memory T cells; Tem, effector memory T cells; Tfh, follicular helper T cells; Trm, tissue resident memory T cells].

A proportion of Tcm cells may also include T follicular helper (Tfh) cells (CXCR-5^+^ Bcl-6^+^) which are critical for B cell antibody responses during infections. These CD4^+^ T cells retain their Tfh cell commitment during the resting memory stage and following reinfection, remain committed to providing help to memory B cells in germinal center (GC) of secondary lymphoid tissues (58, 59). The long-term maintenance of CD4^+^ memory T cell requires expression of IL-7RΑ, which is low on effector cells. CD4^+^ memory T cells have a distinct advantage over effector cells during the contraction phase owing to their ability to respond to IL-7, which stimulates anti-apoptotic bcl-6 expression. CD4^+^ memory T cells are maintained by IL-7 and IL-15, which also support the periodic replenishment of these cells (60) (**Figure 2**). In contrast, IL-2 favors effector CD4^+^ T cell formation (61).

VL generates an organ specific immune response in mice where Th1 cell-mediated responses and granuloma formation contribute to infection resolution in the liver and resistance to reinfection (62). In dogs formation of structured granulomas formation has been associated with disease control, while unstructured granulomas were associated with disease (63). Interestingly, in mice, parasites are controlled in the liver, but they persist and propagate slowly in the spleen (64, 65). Tissue specific immunity can also be observed in human VL, exemplified by nodular post Kala-Azar dermal leishmaniasis (PKDL), where parasites are controlled in the viscera after drug treatment, but continue replicating in the skin (65). This highlights the complexity of immune responses in different anatomical sites following resolution of visceral disease.

Furthermore, the chronic nature of VL, which affects organs such as the spleen, liver, and bone marrow, raises the possibility that prolonged exposure of the immune system to parasitic antigens may disrupt memory T cell development and maintenance, and the emergence of effector T cell populations, making it difficult to conclusively show what happens in the absence of infection.

## Longevity of anti-parasitic immunity

The generation of anti-parasitic immunological memory in a given population occurs when individuals in endemic areas acquire immunity to subsequent infections after recovering from the disease. The cyclic nature of VL, referring to the periodic rise and fall of disease incidence in certain regions (66–68), is influenced by various factors, including the complex interactions between the parasite, the vector, the host population, population demographics, duration and intensity of the cycles and the environment. Importantly, this cyclic course of disease incidence has important implications for long-term immune maintenance. During periods of low disease transmission, asymptomatic individuals and those who have previously developed VL, been treated and recovered, contribute to the pool of immune individuals (64). This acquired immunity helps protect them from future infections. However, there is evidence to suggest that acquired immunity may gradually decline over time at least in CL (69). The duration and strength of immunity can vary among individuals, with some studies indicating that immunity may wane after a few years following the last infection in a given community (70, 71). This decline in immunity can lead to an increase in susceptible individuals in the population, providing opportunities for new infections and potential disease outbreaks during periods of increased transmission. However, repeated exposure to the parasite can lead to the maintenance of memory within a given population, as a significant portion of individuals will retain some level of immunity (69).

The maintenance of protective immunity in populations is also thought to be facilitated by the presence of asymptomatic carriers or individuals with subclinical infections. It is estimated that around 90% of *Leishmania*-infected individuals never present with any clinical symptoms and remain asymptomatic in India (72). However, diverse proportions of asymptomatic infections caused by *L. (L.) donovani* or *L. (L.) infantum* relative to symptomatic clinical cases occur in different part of the world. These ratios have been documented as 1:2.4 in Sudan, 4:1 in Kenya, 5.6:1 in Ethiopia, 6:1 to 18:1 in Brazil, 4:1 in Bangladesh, and 8.9:1 in India and Nepal (1, 73). Asymptomatic individuals, although not displaying clinical symptoms, still harbor parasites and therefore have the potential to contribute to disease transmission (68, 74). A recent xenodiagnoses study showed that asymptomatic individuals were not infectious to sand-fly vectors and may be considered an unimportant contributor to the maintenance of the *L. (L.) donovani* transmission cycle (75).

The treatment of VL is aimed at curing infected individuals and reducing the burden of the disease at the individual level rather than affecting the overall parasite population dynamics and maintenance of the parasite in populations. VL patients generally respond well to treatment, but treatment failure rates can range from 2-7% (76), and may reach up to 60% in HIV/VL co-infected individuals (77). Furthermore, incomplete treatment or treatment failure can lead to the persistence of parasites at a higher level than found in asymptomatic individuals. There is also an increasing concern that parasites will develop resistance to approved chemotherapeutic interventions, which may also promote disease relapse (78). Persisting splenomegaly after treatment may indicate incomplete parasites clearance and be another indicator of treatment failure (79). In addition, the immune status of a VL patient, particularly low CD4^+^ T cell count at the time of diagnosis and treatment, or during follow-ups may indicate a higher risk of relapse (80).

Multiple cases of reinfection in a community could indicate compromised immunity and/or diminished numbers of parasite-specific CD4^+^ memory T cells at the population level. To improve our current treatment and prevention approaches it is important to distinguish between relapsed disease and disease caused by reinfection, as relapse can indicate failure of treatment or persistence of large numbers of *Leishmania* parasites (81). A reinfection differs from a relapse in that new parasites are introduced. Reinfection signifies the lack or loss of infection-induced immunity. The impact of sterile cure following drug treatment (i.e., no parasites remaining) on the maintenance of immunity to VL is not known, and this may be an issue with the use of more effective anti-parasitic drugs such as Ambisome. However, evidence from studies in mice model of CL fuels the idea of concomitant immunity (82, 83)

In areas where the disease has been eliminated, the reintroduction of parasites can cause new disease outbreaks. Hence, monitoring immunity in regions where VL elimination programs have occurred or are ongoing could be informative in regard to population vulnerability to infection. Similar issues have been raised in malaria control programs, where continuous exposer to parasites may be important to maintain protective immunity in the population (84, 85).

## Effect of HIV co-infections on T cell responses

The co-infection of *Leishmania* and HIV presents significant challenges for disease control and elimination efforts. Individuals with HIV have an increased risk of contracting VL, and VL can accelerate the progression of AIDS by promoting viral replication (6). HIV infection induces immune suppression primarily through the depletion of CD4^+^ T cells, which play a crucial role in controlling the *Leishmania* parasite. Additionally, the chronic immune activation caused by the *Leishmania* parasite may benefit HIV replication. This prolonged immune stimulation not only supports viral replication but also increases the expression of CCR5, a co-receptor crucial for HIV entry, on both CD4^+^ and CD8^+^ T cells (6, 86, 87). Various viral and parasitic factors contribute synergistically to promote each other’s persistence (88).

Patients co-infected with VL and HIV serve as critical reservoirs for *Leishmania*, with a higher potential for transmission compared to HIV-negative VL patients (89, 90). This heightened risk is due to the difficulty in fully eradicating the parasite in immunocompromised individuals (91), allowing *Leishmania* to persist and spread within populations. Furthermore, co-infected patients exhibit higher rates of relapse, as reported in multiple studies (92, 93).

## Effect of nutritional status on T cell responses

The nutritional status of the host is a critical factor influencing immune responses and affecting the course and severity of VL. Malnutrition has been strongly linked to increased VL susceptibility, which is often associated with the socioeconomic conditions of endemic regions (94, 95). In a recent study, poly-nutrient-deficient *L. infantum*-infected mice exhibited higher parasite burdens in the spleen and liver, reduced T lymphocyte counts with lower IFN-γ, and elevated IL-10 production (96). Besides proteins, carbohydrates, and lipids, adequate vitamins and minerals (e.g., calcium, iron, iodine) are essential for adaptive and innate immunity, influencing treatment outcomes and relapse risk in leishmaniasis (97).

## Factors affecting parasite persistence

**Figure 3:**
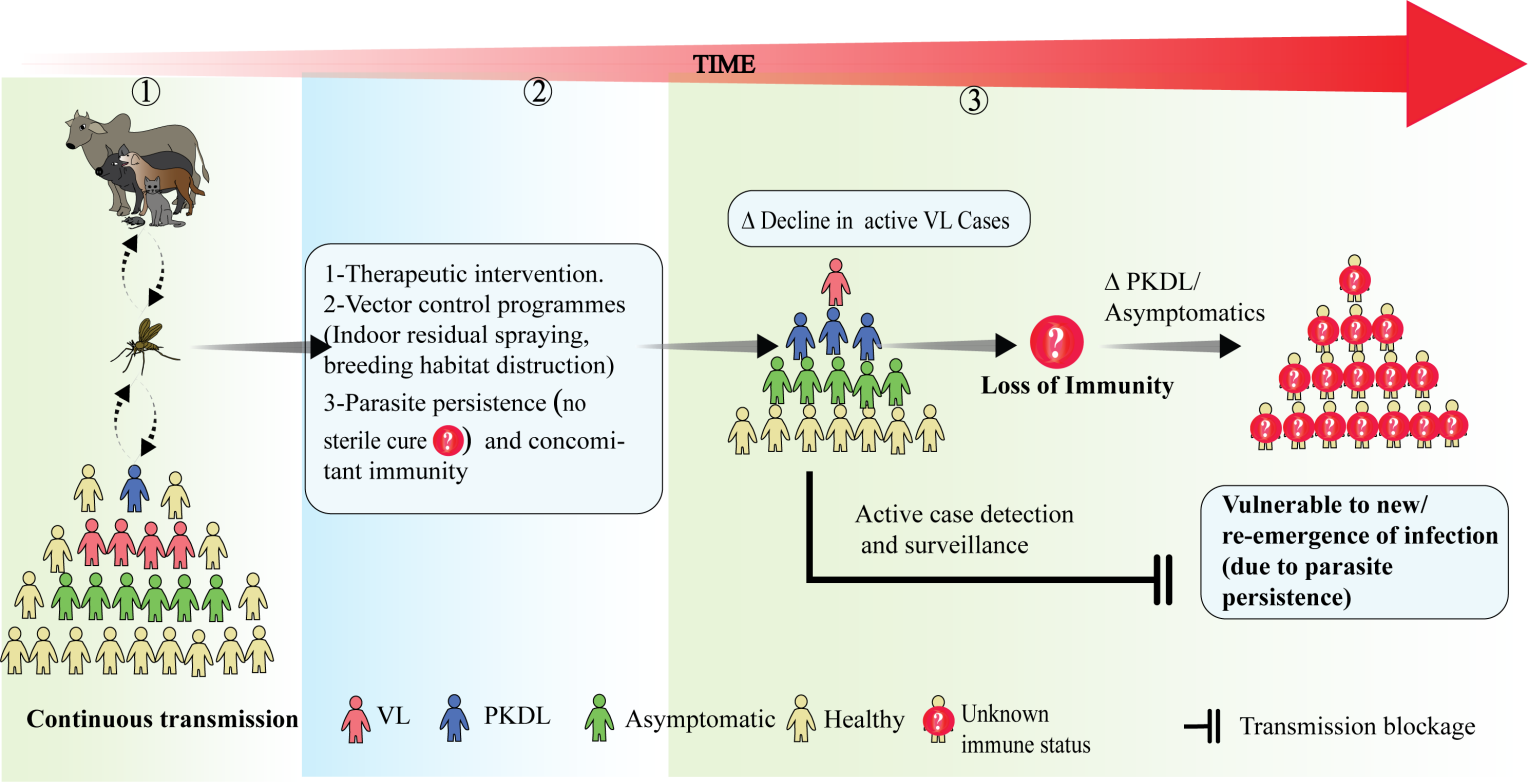
The effect of disease transmission on the maintenance of long-term population immunity and its implications for disease dynamics: (1) During continuous transmission in an endemic area there is a constant flow of leishmanial antigen among humans and other reservoir hosts and people with active VL, PKDL and asymptomatic infection co-exist. (2) Following disease & transmission control measures such as treatment interventions and vector control programs, parasite transmission may decline along with the number of active VL cases. However, parasites may still persist in treated VL cases, maintaining concomitant immunity. (3) An overall decline in transmission raises the possibility for impacts on long term population immunity. If this declines, it is important to understand the roles played by asymptomatic and PKDL individuals in the maintenance of population-level immunity and possible disease outbreaks. Factors effecting the vulnerability of populations to new/re-emergences of infection are still largely unknown. Thus, active case detection and surveillance still remains the best option to predict future outbreaks.

As mentioned above, the continued exposure to parasite antigen (concomitant immunity), is likely important for maintaining effective anti-parasitic immunological memory. However, other disease interventions and control measures can impact on this exposure, and must be considered when managing the potential for disease outbreaks. Governments and organizations striving to eradicate VL have faced significant obstacles despite their substantial efforts, and the difficulties posed by the disease’s biology and its intricate socioeconomic association makes this task considerably more difficult. The disease has a cyclic nature in India as predicted before the epidemics of 1977 and 1991 by Napier (98, 99). The known cycle period of 10-15 years in India is not understood, but has been suggested to be associated with sand fly resistance to insecticides (100), which supports the implementation of vector control programs in conjunction with active case detection and treatment interventions (**Figure 3**). There have been times in the past when the disease appeared to vanish from highly endemic areas, as was the case in Assam (India), where an epidemic occurred between the late 19th and mid-20th century, killing thousands of people. However, the disease disappeared in the following years (31). At the time, credit for this was given to the use of DDT in the malaria elimination program. As a result of this effort, sand fly vectors were almost completely eliminated, and transmission ceased. However, there was an escalation in number of cases, nearly 60 years later, and epidemics reoccurred in 2004 and 2008, indicating a resurgence of vectors due to insecticide resistance or inefficient vector control strategies (101). Various environmental factors influence parasite life cycle and their persistence in the environment. For example, VL is associated with sub-standard housing and climatic conditions such as high humidity and temperature. A focus on the control of breeding habitats for sand flies can greatly reduce transmission rates (102, 103). The fact that there a range of reservoir hosts, which include rodents, wild dogs and even domestic dogs and cattle (104), indicate that the parasites are continually present in the immediate environment of humans, as long as sand flies are present to transmit them. Reports suggest that dogs serve as an important reservoir for VL-causing parasites in the Mediterranean and South America (105, 106).

Human-made environmental changes like deforestation, settling near a forest or agriculture practices plays an important role in parasite transmission cycles by promoting closer proximity between humans and non-human reservoir hosts than would normally be the case (107). Even after efforts to limit the breeding or spread of sand fly vectors, active surveillance to identify human reservoirs such as asymptomatic individuals and PKDL patients is necessary (108–111). However, it should be acknowledged that the total eradication of the vector or the parasite may impair immunological memory developed during ongoing transmission and this may contribute to future outbreaks when infected individuals move into an area and/or sand fly vectors are reintroduced (112, 113). Furthermore, there have been suggestions that environmental factors may cause increased infectivity of parasites through genetic modifications resulting in parasite fitness gains (114). These findings highlight the need to understand the presence of parasites in environments and how this changes, as well as how immunity is maintained and the factors contributing to outbreaks (**Figure 3**).

## PKDL and the challenge of persisting parasites

PKDL is a cutaneous manifestation of *L. (L.) donovani* infection that can arise after what appeared to be successful drug treatment. In PKDL patients, the immune system fails to control parasite growth in skin, while an effective systemic response protects the viscera. This suggests the presence of type 2 immune responses or other types of regulatory responses in the skin (115). Moreover, systemic protective immunity that persists is likely maintained by antigen-experienced memory cells, as evidenced by cytokine and proliferative CD4^+^ T cell responses during whole blood assay (56, 57). However, skin-specific immunological anomalies do exists, which could be attributed to a lack of organ-specific CD4^+^ T cell memory (116). PKDL presents with macules, papules or nodules on skin and rarely occurs in asymptomatic individuals having had no signs of VL. The disease is frequently seen before and after cure of African VL, while in India, PKDL is rare and the onset generally occurs years after VL cure. Notably, the African form is typically self-healing while the Indian form of disease is chronic. In nodular PKDL, the presence of heavily infected macrophages in skin can serve as reservoirs and facilitate transmission, posing a threat to the VL elimination programs (117). Pathogenesis of PKDL is considered to be immunologically mediated with increased IL-10 production by keratinocytes and other cells having been reported during active disease and has been suggested to promote the parasite persistence (118). The mRNA profiles from both blood and skin indicated that PKDL patients have a mixed Th1 and Th2 cell response, as indicated by high levels of expression of IFNγ, TNF, TGFβ, IL-10, IL-6, and IL-4 with decreased mRNA expression of IFN-γ receptor 1 (IFNγR1) (119). Accumulating evidence suggest a role for IL-10 mediated Treg cell differentiation and accumulation in the skin associated with disease severity (120). Understanding why some individuals develop PKDL is a major challenge in the context of VL elimination. Understanding how protection of visceral organs is maintained while disease manifests in the skin is an intriguing challenge, and understanding the immunological mechanisms responsible may shed important light about how immunity to VL is maintained and/or lost.

## CD4^+^ T cell memory response and vaccine development

The idea that a vaccine to protect against VL would be feasible arose from the past success of “Leishmanization” where live parasites from cutaneous lesions were used to cause cutaneous disease in covered body locations to protect against disfiguring CL (28, 121–123). This form of deliberate infection typically conferred life-long protection against subsequent disease (124). It was relatively effective in terms of the protection it offered, but due to potential complications in some individuals, this vaccination technique was gradually abandoned (125). The creation of a vaccine against VL has still not been achieved and a major bottle neck is our incomplete understanding about how protective, anti-parasitic CD4^+^ T cell memory is generated and maintained in humans. However, the development of VL vaccines for dogs, like Leish-111f + MPL-SE (126), offers hope that vaccines for humans can be developed (127). Murine studies utilizing different vaccine candidates have also shown that a potent memory T cell response can be generated following immunization that can confer long term protection against VL (128, 129). The prospect of developing a successful VL vaccine is further strengthened by reports suggesting existence of leishmanial antigen reactive memory T cells long after clinical cure of human infection (49, 57).

CD4^+^ memory T cells are crucial for the development of an effective vaccine against VL as they are central to orchestrating the adaptive immune response needed to control and eliminate *Leishmania* parasites. These cells facilitate the production of cytokines, such as IFN-γ, which activate macrophages to effectively kill intracellular parasites (130). Moreover, CD4^+^ memory T cells enable a rapid and robust immune response upon re-exposure to the pathogen, thereby ensuring long-term immunity (131). Research has shown that polyclonal expansion of effector memory (Tem) cells occurs after treatment in human VL cases (132). Vaccine studies have also linked the generation of CD4^+^ T cell memory with protective immunity (133). The persistence of these memory T cells after initial infection or vaccination is vital for providing lasting protection, evidenced by the induction of tissue-resident memory (Trm) cells in mouse models of cutaneous leishmaniasis (CL) (134). Studies performed in both murine and canine models found that effective cell mediated immunity (CMI) along with Tcm (CD127^high^ CD44^high^ CD45^low^ CD62L^high^ CD197^high^) and Tem (CD62L^low^ CD44^high^ CD127^high^ CD197^low^) cells not necessarily require live parasites and could be generated by a peptide cocktail from *L. (L.) infantum*, resulting in a decreased parasite load in splenic cell cultures of BALB/c mice when reinfected (135). A similar approach using an immunodominant fusion of multiple peptide epitopes from *L. (L.) major, L. (L.) donovani* and *L. (V.) brazilinesis* species was also able to generate potent CMI and CD4^+^ memory T cells, resulting in protection following challenge with *L. (L.) infantum* infection (136). CD4^+^ memory T cell generation against *Leishmania* parasites, just like other diseases, not only depends upon antigen stimulation, co-stimulation and DC cytokine production, but is also influenced by other immune cells. For example, marginal zone B cells (MZB) were reported to impair CD8^+^ and CD4^+^ Tem cell generation against *L. (L.) donovani* challenge in mice (137).

Interestingly, experimental models using *L. (L.) infantum* infection indicate that persistent parasite may negatively affect vaccine induced frequencies of effector and memory CD4^+^ T cells, as well as CD8^+^ T cells, in comparison to the non-infected mice. However when parasites where killed by drug treatment this resulted in release of antigens and the vaccine induced memory T cell development increased upon re-exposure to antigen (138).

The use of LACK (leishmania homologue for receptors of activated C kinase), an intracellular *Leishmania* protein as a vaccine has been shown to confer protection against CL in murine models (139) and against VL in dogs (140). The protection was reported to be mediated by a Th1 type immune responses. Another vaccination study identified the immune parameters involved in protective immunity against CL in BALB/c mice. The study reported that CD4^+^ and CD8^+^ effector memory T cells, along with TNF, IFNγ, and other type 1 cytokines, play crucial role in protection (141). Similarly, the polyprotein subunit vaccine KSAC produced by fusing the antigenic proteins KMP11, SMT, A2, and CPB has been shown to provide protection mediated by IFN-γ-producing Th1 cells (142).

The presence of leishmanial antigen-reactive CD4^+^ memory T cells long after clinical recovery highlights their role in immune surveillance and protection against reinfection. *Leishmania* infection can induce partial immunity, marked by memory T cells ready to rapidly respond upon re-exposure. Individuals recovered from symptomatic VL often display a more robust, IFN-γ-dominant cytokine response *ex vivo*, which may contribute to enhanced resistance to severe disease upon re-infection (143, 144). In mouse model of CL, persistent low-level parasites are believed to confer protection by maintaining immunological memory (18). However, evidence of this persistence in human cases of CL and VL is limited, as most studies focus on model organisms. Additionally, chronic exposure to leishmanial antigens can lead to T cell unresponsiveness, raising concerns about the effectiveness of these memory T cells over time. Chronic exposure to leishmanial antigens can also lead to regulatory T cell induction, which may compromise T cell responses (34, 145). These immune adaptations present challenges for vaccination efforts, as immune tolerance or exhaustion can reduce vaccine efficacy in VL endemic populations, similar to observations in populations with chronic *Plasmodium* exposure (146). Consequently, studying immune responses in asymptomatic individuals or VL patients that have been successfully drug treated may provide guidance on biomarkers that can be used to assess the effectiveness of vaccination and the duration of subsequent immunity.

## Discussion and future prospective

Leishmaniasis is a neglected tropical disease and there is a critical need for an effective vaccine and more potent pharmacological therapies. Most of our knowledge regarding the immunological aspects of VL comes from experimental models and a lack of proper insight from infected humans has resulted in a major knowledge gap. To understand why those who have been treated gain protective immunity and why the majority of infected individuals do not develop disease, will require studies on human immune responses. Regarding VL, while it is evident that a Th1 cell immune response confers protection against the disease, we still don’t know whether the persistence of parasites is needed to maintain protective CD4^+^ T cell memory responses. Reports suggesting the persistence of low parasite numbers is needed for protective immunity has been reported in mouse models (reviewed in (20). Concomitant immunity, in which immunity to pathogens relies on viable pathogen persistence, may nonetheless, hold the key to delaying the onset of disease, but relapses might occur with lifelong persistence of parasites (138). There is an important need to establish the longevity of the CD4^+^ memory T cell pool following sterile cure, as this will help determine the degree of disease susceptibility in areas of potential disease outbreaks.

Finally, it is worth highlighting again that people who recover from VL following drug treatment are protected against future disease, thus demonstrating there is a natural mechanism of protective immunity that needs to be understood so we can design vaccines and treatments to eliminate this disease.
